# Simultaneous Inhibition of Histone Deacetylases and RNA Synthesis Enables Totipotency Reprogramming in Pig SCNT Embryos

**DOI:** 10.3390/ijms232214142

**Published:** 2022-11-16

**Authors:** Mariana Priotto de Macedo, Werner Giehl Glanzner, Karina Gutierrez, Luke Currin, Vanessa Guay, Maria Elena Carrillo Herrera, Zigomar da Silva, Hernan Baldassarre, Serge McGraw, Vilceu Bordignon

**Affiliations:** 1 Department of Animal Science, McGill University, Sainte-Anne-de-Bellevue, QC H9X 3V9, Canada; 2 CHU Sainte-Justine Research Center, Université de Montréal, Montréal, QC H3T 1C5, Canada

**Keywords:** SCNT, cell reprogramming, HDACi, DRB, porcine

## Abstract

Combining somatic cell nuclear transfer (SCNT) with genome editing technologies has emerged as a powerful platform for the creation of unique swine lineages for agricultural and biomedical applications. However, successful application of this research platform is still hampered by the low efficiency of these technologies, particularly in attaining complete cell reprogramming for the production of cloned pigs. Treating SCNT embryos with histone deacetylase inhibitors (HDACis), such as Scriptaid, has been routinely used to facilitate chromatin reprogramming after nuclear transfer. While increasing histone acetylation leads to a more relaxed chromatin configuration that facilitates the access of reprogramming factors and DNA repair machinery, it may also promote the expression of genes that are unnecessary or detrimental for normal embryo development. In this study, we evaluated the impact of inhibiting both histone deacetylases and RNA synthesis on pre- and post-implantation development of pig SCNT embryos. Our findings revealed that transcription can be inhibited for up to 40 h of development in porcine embryos, produced either by activation, fertilization or SCNT, without detrimentally affecting their capacity to form a blastocyst and their average number of cells at this developmental stage. Importantly, inhibiting RNA synthesis during HDACi treatment resulted in SCNT blastocysts with a greater number of cells and more abundant transcripts for genes related to embryo genome activation on days 2, 3 and 4 of development, compared to SCNT embryos that were treated with HDACi only. In addition, concomitant inhibition of histone deacetylases and RNA synthesis promoted the full reprograming of somatic cells, as evidenced by the normal fetal and full-term development of SCNT embryos. This combined treatment may improve the efficiency of the genome-editing + SCNT platform in swine, which should be further tested by transferring more SCNT embryos and evaluating the health and growth performance of the cloned pigs.

## 1. Introduction

Pigs are not only extremely important for food production, but also as animal models for biomedical research, due to their physiological and anatomical similarities to humans [1]. Consequently, there has been growing interest in pig somatic cell nuclear transfer (SCNT), which, combined with genome editing technologies, represent an important platform for the creation of unique animal models for the study of diseases or xenotransplantation. SCNT has relevant applications in biomedicine (e.g., for the generation of unique animal models), agriculture (e.g., for the replication of superior livestock) and conservation (e.g., for the replication of endangered species). However, after many years since it was first shown that differentiated somatic cells can be used to clone mammals [2], important obstacles for increasing SCNT efficiency remain to be overcome. Following SCNT, it is expected that factors present in the cytoplasm of the oocyte can reprogram the epigenetic status of the donor cell to a zygotic state, which allows the somatic donor cell to reacquire totipotency [3]. Other methods used to reprogram porcine somatic cells include the expression of transcription factors [4,5,6] and exposure to cytoplasmic extracts of porcine oocytes [7,8]. However, cell reprogramming efficiency by these methods and chimeric contribution of in vitro reprogramed cells remain very low. Moreover, the mechanisms responsible for reprogramming following SCNT are still not fully understood. While new discoveries in the field of cellular reprogramming have come a long way, its translation into practical protocols for improving SCNT efficiency has not progressed at the same pace.

Many approaches have been tested to overcome reprogramming roadblocks in SCNT embryos, such as chromatin and epigenetic regulators, which would create a more favorable environment for reprogramming [9]. Cellular reprogramming and totipotency acquisition are regulated by epigenetic modifications such as histone acetylation, as well as histone and DNA methylation [10,11]. For example, genes involved in post-translational modifications and regulation of the embryo genome activation (EGA), such as histone demethylases (*KDMs*), DNA methyltransferases (*DNMTS*) and transcription factors (e.g., *DUXA*, *DPPA2*, *DPPA4*), were noticed to be abnormally expressed in SCNT embryos [12,13,14]. In addition, SCNT embryos were shown to have lower histone acetylation levels compared with embryos produced by in vitro fertilization (IVF) [15]. Thus, increasing histone acetylation by inhibiting histone deacetylases has been used to improve SCNT efficiency in several species, including pigs [9,16,17,18,19]. Higher histone acetylation levels are related to a more open and permissive chromatin state, through reduction of the interaction between histones and DNA [20], which allows transcriptional activation and a better access of reprogramming factors to the chromatin. Scriptaid and Trichostatin A (TSA) are the more commonly used histone deacetylase inhibitors (HDACi) to improve cell reprogramming after SCNT in pig embryos [9]. Scriptaid seems to have lower cellular toxicity and promotes higher acetylation levels compared to other HDACi compounds [15,21]. Scriptaid treatment was shown to increase blastocyst rates [18], decrease apoptosis [22], improve DNA damage repair [23] and improve full-term development of SCNT pig embryos [15,24].

The relaxed and more permissive chromatin state caused by HDACi is thought to facilitate the proper activation of the embryonic genome, which is critical for the successful development of SCNT embryos [25,26]. However, both excessive gene silencing and activation have been correlated with failure in cell reprogramming following SCNT [27,28]. Indeed, persistent somatic transcripts were detected in induced pluripotent stem cells [29], as well as in SCNT embryos [30]. Thus, it is possible that exposure to HDACi after SCNT may not only promote an opening chromatin state that facilitate the access of reprogramming factors, but it may also favor the continued expression of somatic genes from the donor cells, which could affect normal embryo cell functions. Therefore, inhibiting RNA synthesis while increasing histone acetylation may represent a new strategy to improve cell reprogramming, normal cell function and development of SCNT embryos.

In a previous study with bovine SCNT, we provided evidence that treatment of embryos for 15 h after nuclear transfer with a combination of Scriptaid and 5,6-Dichlorobenzimidazole 1-β-D-ribofuranoside (DRB), a reversible inhibitor of RNA polymerase II, enhanced chromatin remodeling, prevented the premature expression of several transcripts encoding important epigenetic regulators and increased the total number of cells per blastocyst [31]. On the other hand, the exposure of mouse embryos to DRB for 4 to 20 h following fertilization significantly impaired embryo development, which indicates that early transcription is essential for normal development in this species [32]. Therefore, it is possible that these strategies for enhancing cell reprogramming in SCNT based on transcriptional inhibition can only be implemented in species where the EGA occurs at later stages of development. It is also possible that inhibiting the transcriptional activity has a different impact on fertilized and SCNT embryos. Thus, the goals of this study were to determine how long the transcriptional activity can be inhibited in porcine embryos produced by parthenogenetic activation (PA), in vitro fertilization (IVF) and SCNT without affecting their developmental capacity and quality, and to investigate if the simultaneous inhibition of RNA synthesis and histone deacetylases is a viable strategy to improve cell reprogramming and development of pig SCNT embryos.

## 2. Results

### 2.1. How Long Can Transcription Be Inhibited in Pig Embryos without Affecting Their Development?

**Figure 1:**
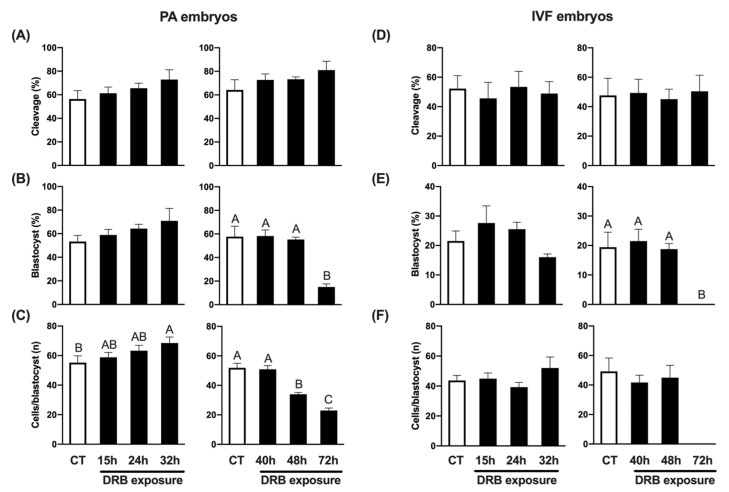
Effect of exposure to DRB for different times on development of PA and IVF embryos. (**A**) Cleavage rates of PA embryos treated with DRB for 0 h (CT, n = 152), 15 h (n = 151), 24 h (n = 152) and 32 h (n = 153) (left graphic), or 0 h (CT, n = 198), 40 h (n = 195), 48 h (n = 194) and 72 h (n = 197) (right graphic). (**B**) Blastocyst rates of PA embryos treated with DRB for 0 h (CT, n = 88), 15 h (n = 95), 24 h (n = 100) and 32 h (n = 97) (left graphic), or 0 h (CT, n = 125), 40 h (n = 141), 48 h (n = 142) and 72 h (n = 157) (right graphic). (**C**) Average number of cells per blastocyst in PA embryos treated with DRB for 0 h (CT, n = 47), 15 h (n = 57), 24 h (n = 63) and 32 h (n = 66) (left graphic), or 0 h (CT, n = 72), 40 h (n = 82), 48 h (n = 79) and 72 h (n = 24) (right graphic). (**D**) Cleavage rates of IVF embryos treated with DRB for 0 h (CT, n = 275), 15 h (n = 281), 24 h (n = 267) and 32 h (n = 279) (left graphic), or 0 h (CT, n = 215), 40 h (n = 212), 48 h (n = 216) and 72 h (n = 213) (right graphic). (**E**) Blastocyst rates of IVF embryos treated with DRB for 0 h (CT, n = 148), 15 h (n = 135), 24 h (n = 149) and 32 h (n = 141) (left graphic), or 0 h (CT, n = 99), 40 h (n = 102), 48 h (n = 99) and 72 h (n = 109) (right graphic). (**F**) Average number of cells per blastocyst in IVF embryos treated with DRB for 0 h (CT, n = 33), 15 h (n = 32), 24 h (n = 40) and 32 h (n = 22) (left graphic), or 0 h (CT, n = 21), 40 h (n = 21), 48 h (n = 18) and 72 h (n = 0) (right graphic). For cleavage results, “n” refers to number of activated/fertilized oocytes per group; for blastocysts results, “n” refers to number of cleaved embryos per group; and for cells/blastocyst results, “n” refers to number of blastocysts analyzed per group. Different letters indicate statistical differences between DRB exposure times within each experiment (*p* < 0.05).

To explore if transcriptional inhibition using DRB was both effective and reversible, we exposed porcine embryos produced by either PA or IVF to 100 µM DRB for different lengths of time (15 h, 24 h, 32 h, 40 h, 48 h or 72 h), and then transferred them to standard culture conditions until day 7 to evaluate blastocyst development rates and count the total number of cells per blastocyst. Because it was not possible to test all the groups in the same experiment, due to the large number of oocytes required, the study was conducted in two phases. First, control embryos (CT, not treated with DRB) were compared to DRB-treated embryos for 15 h, 24 h or 32 h, and second, CT embryos were compared to DRB-treated embryos for 40 h, 48 h or 72 h. The concentration of DRB was chosen based on previous experiments by our group [31], and the efficacy of transcriptional inhibition was confirmed by incubating PA embryos for 7 days in the presence of DRB, which completely blocked embryo development to the blastocyst stage (Supplementary Figure S1A). To further confirm DRB efficacy on transcriptional inhibition, porcine fetal fibroblasts (PFF) were cultured for 15 h in the presence of DRB, Scriptaid + DRB, Scriptaid alone or not treated (CT cells), and their transcriptional activity was evaluated by assessing 5-Ethynil Uridine (EU) incorporation. While CT and Scriptaid-treated cells showed a strong fluorescent signal for EU incorporation, those treated with DRB or Scriptaid + DRB showed a weaker EU signal, which confirmed the efficacy of DRB in preventing new RNA synthesis (Supplementary Figure S1B). We observed that embryo cleavage rates, assessed at 48 h of culture, were not affected by DRB treatment in either PA (Figure 1A) or IVF (Figure 1D) embryos. Embryo development to the blastocyst stage was not significantly affected by culture in the presence of DRB for up to 48 h of development, but blastocyst rates were dramatically decreased (~3 fold) in PA (Figure 1B), and totally inhibited in IVF embryos (Figure 1E) that were exposed to DRB for the first 72 h of culture. The average cell number per blastocyst was significantly lower in PA embryos treated with DRB for either 48 h or 72 h (Figure 1C), but it was not statistically different between IVF CT and DRB-treated embryos for 48 h (Figure 1F). Interestingly, shorter exposure to DRB tended to increase total cell numbers in PA blastocysts, as evidenced by the significantly higher number of cells in embryos that were exposed to DRB for 32 h compared to CT embryos (Figure 1C). These results demonstrated that transcriptional inhibition with DRB for up to 40 h in PA and IVF pig embryos is not only compatible with normal development to the blastocyst stage but also results in blastocysts with a normal number of cells.

### 2.2. Impact of Transcriptional Inhibition on Development of SCNT Embryos

**Figure 2:**
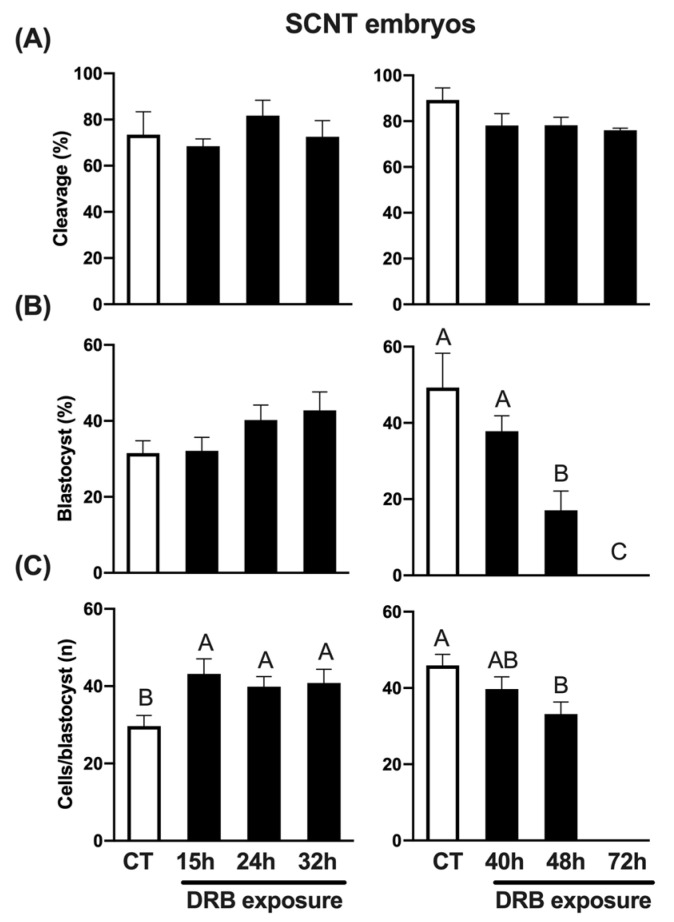
Effect of exposure to DRB for different times on early development of SCNT embryos. (**A**) Cleavage rates of SCNT embryos treated with DRB for 0 h (CT, n = 79), 15 h (n = 85), 24 h (n = 76) and 32 h (n = 79) (left graphic), or 0 h (CT, n = 92), 40 h (n = 94), 48 h (n = 97) and 72 h (n = 94) (right graphic). (**B**) Blastocyst rates of SCNT embryos treated with DRB for 0 h (CT, n = 58), 15 h (n = 58), 24 h (n = 62) and 32 h (n = 58) (left graphic), or 0 h (CT, n = 81), 40 h (n = 74), 48 h (n = 74) and 72 h (n = 71) (right graphic). (**C**) Average number of cells per blastocyst in SCNT embryos treated with DRB for 0 h (CT, n = 19), 15 h (n = 19), 24 h (n = 25) and 32 h (n = 25) (left graphic), or 0 h (CT, n = 41), 40 h (n = 29), 48 h (n = 13) and 72 h (n = 0) (right graphic). CT SCNT embryos were exposed to Scriptaid for 15 h and DRB-treated embryos were exposed to Scriptaid + DRB for 15 h, and then only DRB for the remaining times of treatment. For cleavage results, “n” refers to number of activated cloned oocytes per group; for blastocysts results, “n” refers to number of cleaved embryos per group; and for cells/blastocyst results, “n” refers to number of blastocysts analyzed per group. Different letters indicate statistical differences between DRB exposure times within each experiment (*p* < 0.05).

To assess the impact of DRB in SCNT embryos, control (CT) embryos were treated with Scriptaid for 15 h, while treated embryos were exposed to both Scriptaid and DRB for 15 h, and then only DRB for up to 24 h, 32 h, 40 h, 48 h and 72 h following nuclear transfer and oocyte activation. Because of the large number of embryos required for all the treatments, the experiment was conducted in two phases, as described for the previous experiments. Cleavage rates were not affected by DRB treatment (Figure 2A). Blastocyst rates tended to increase in embryos exposed to DRB for up to 32 h but were not statistically different than CT embryos exposed to Scriptaid only (Figure 2B). However, longer exposures to DRB either decreased (48 h) or completely inhibited (72 h) blastocyst formation (Figure 2B). Moreover, exposure to DRB for 48 h significantly decreased blastocyst cell number compared to CT embryos (Figure 2C). Interestingly, the average number of cells per blastocyst was significantly higher than CT in embryos treated with DRB for 15 h, 24 h or 32 h after nuclear transfer and oocyte activation (Figure 2C). These results indicate that concomitantly inhibiting histone deacetylase and RNA synthesis for 15 h and then only inhibiting RNA synthesis for up to 32 h may facilitate the resetting of the somatic chromatin and improve cell functions in pig SCNT embryos.

### 2.3. Transcriptional Activity in SCNT Embryos That Were Treated with Histone Deacetylase and RNA Synthesis Inhibitors

**Figure 3:**
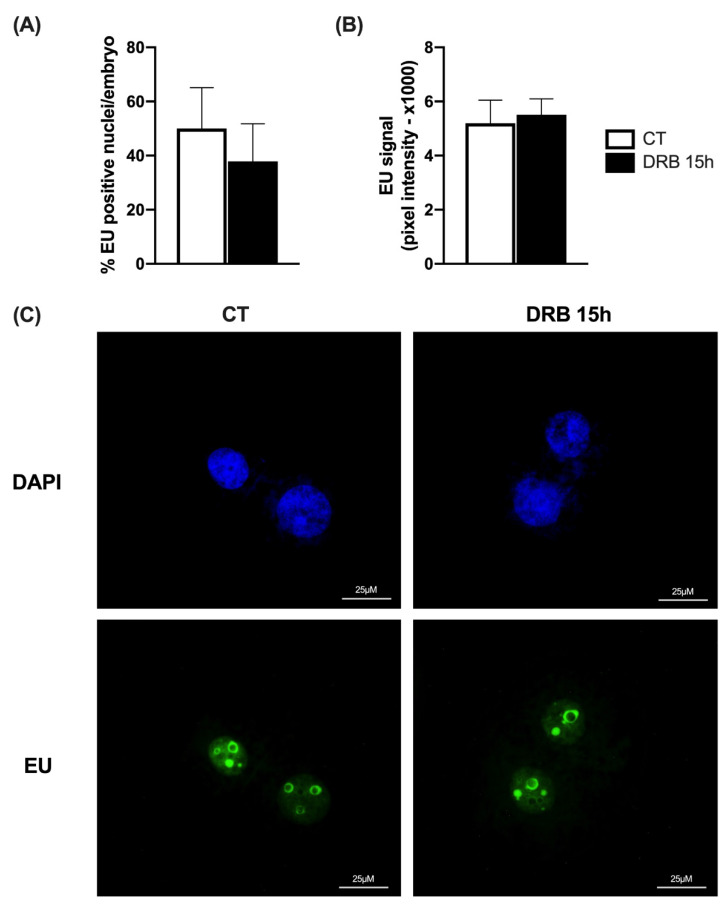
Assessment of new RNA synthesis by EU incorporation on D3 SCNT embryos. (**A**) Proportion of nuclei per embryo with positive staining for EU in SCNT embryos treated for 15 h with Scriptaid only (CT, n = 12) or Scriptaid + DRB (DRB, n = 12). (**B**) Pixel intensity for EU incorporation in CT (n = 12) and DRB (n = 12) nuclei. (**C**) Representative fluorescent images showing DAPI and EU staining. There was no statistical difference between CT and DRB groups.

Since 15 h of exposure to DRB was the first time point to improve total cell number in SCNT blastocysts, we chose this timepoint to evaluate if the transcriptional inhibition after nuclear transfer would affect the activation of transcriptional activity in the developing SCNT embryos. For this, we first assessed new RNA synthesis by quantifying EU incorporation on day 3 of embryo development after SCNT. We observed that both the proportion of nuclei showing a positive fluorescent signal for EU incorporation (Figure 3A,C) and the EU fluorescence intensity (Figure 3B,C) were not statistically different between CT and DRB-treated embryos for 15 h. These findings indicated that the onset of transcriptional activity in pig SCNT embryos was not delayed by 15 h of DRB treatment after nuclear transfer.

**Figure 4:**
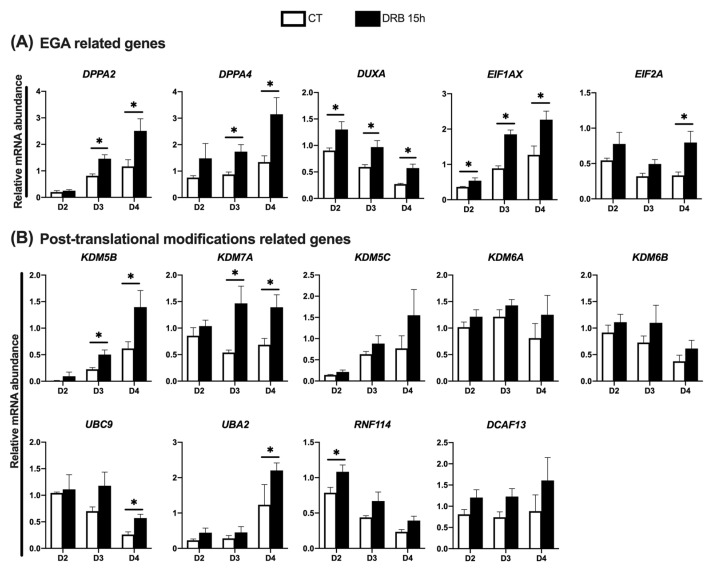
Relative abundance of transcripts at different days of development in SCNT embryos that were treated with Scriptaid (CT) or Scriptaid + DRB for 15 h after nuclear transfer. (**A**) Genes involved in the EGA and (**B**) gene involved in post-translational modifications. A total number of 10 to 15 embryos were collected per replicate (n = 3) and treatment for each day of development. Asterisks indicate statistical difference between treatments in the same day of development (* *p* < 0.05).

We then investigated if DRB treatment affected the relative mRNA abundance of genes regulated during EGA (Figure 4A), as well as genes involved in the regulation of important post-translational modifications (Figure 4B). For this, cDNA samples produced from CT and DRB-treated SCNT embryos on days 2, 3 and 4 of development were used for qPCR analyses. Regarding EGA related genes, DRB treatment increased the relative mRNA abundance of DUXA and EIF1AX on D2, D3 and D4 embryos; DPPA2 and DPPA4 on D3 and D4 embryos; and EIF2A on D4 embryos, compared to CT embryos at the same day of development (Figure 4A). Regarding genes involved in post-translational modifications, DRB treatment increased the mRNA levels of KDM5B and KDM7A on D3 and D4 embryos, UBC9 and UBA2 on D4 embryos, and RNF114 on D2 embryos, compared to CT embryos at the same day of development (Figure 4B). These observations suggest that blocking RNA synthesis during HDACi treatment after nuclear transfer enhanced the transcription of important genes at later stages of development of pig SCNT embryos.

**Figure 5:**
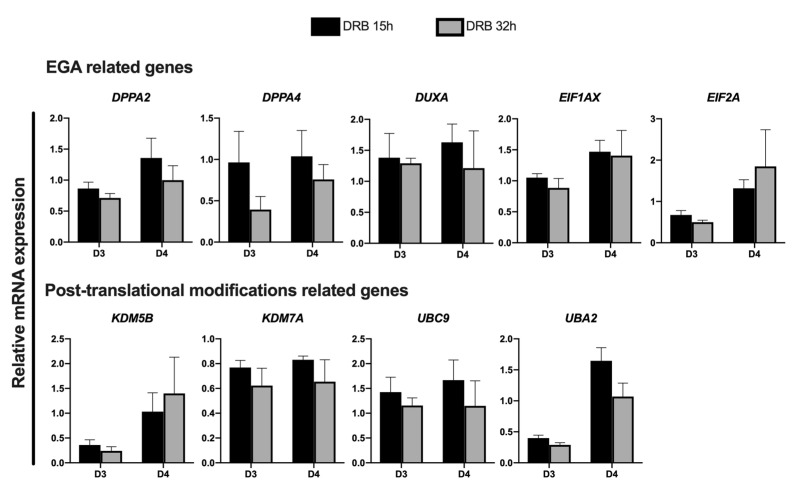
Relative abundance of transcripts in SCNT embryos on days 3 and 4 of development that were treated with Scriptaid for 15 h + DRB for 15 h (DRB 15 h) or 32 h (DRB 32 h) after nuclear transfer. A total number of 10 to 15 embryos were collected per replicate (n = 3) and treatment for each day of development. No statistical difference was observed between treatments at both days of development.

Next, we investigated if extending DRB treatment until 32 h post nuclear transfer would further improve gene expression in SCNT embryos. For this, genes that had their transcription levels increased in embryos treated with DRB for 15 h were compared with embryos treated with DRB for 15 h or 32 h on D3 and D4 of development. The mRNA abundance of some genes tended to be lower in embryos treated with DRB for 32 h; however, there was no statistical difference between embryos treated with DRB for 15 h or 32 h after nuclear transfer (Figure 5). These results indicated that 15 h of DRB treatment is enough to help reset the transcriptional activity and improve the quality of pig SCNT embryos.

### 2.4. Post-Implantation Development of SCNT Embryos Treated with Histone Deacetylase and RNA Synthesis Inhibitors

The last goal in this study was to evaluate if SCNT embryos treated with DRB and Scriptaid for 15 h post nuclear transfer can implant and develop to term upon transfer to recipient gilts. SCNT embryos were produced using three different porcine fetal fibroblast (PFF) cell cultures (PFF A, B and C) and then split into two groups, which were either treated with Scriptaid alone or Scriptaid + DRB for 15 h. Embryos were then surgically transferred to the oviduct of recipient gilts. Pregnancies were confirmed by transabdominal ultrasonography approximately 4 weeks post embryo transfer and then monitored biweekly. Pregnancies from PFF A were interrupted for research purposes and fetuses were collected by cesarian sections. Pregnancies from the other PPF were allowed to term. For SCNT embryos produced using the PFF A cells, the two gilts that were transferred with Scriptaid + DRB-treated embryos became pregnant and 10 fetuses with normal size and morphology were recovered approximately 60 days after embryos transfer, while the two gilts that received embryos that were treated with Scriptaid alone did not become pregnant (Table 1). For SCNT embryos produced using the PFF B cells, pregnancies were confirmed in all four recipients (two per treatment), and one from each treatment carried the pregnancy to term and delivered live cloned piglets, four from the gilt transferred with Scriptaid treated embryos and one from the gilt transferred with Scriptaid +DRB-treated embryos (Table 1). For the PFF C cells, only embryos treated with Scriptaid + DRB for 15 h were transferred to two recipient gilts, both of which became pregnant, carried the pregnancy to term and delivered six cloned piglets, which were all alive and healthy. These results confirmed that treating SCNT pig embryos with both Scriptaid and DRB for 15 h following nuclear transfer is compatible with normal fetal development and production of healthy cloned piglets; however, additional embryo transfers are required to determine if this treatment increases SCNT efficiency in pigs and other species.

## 3. Discussion

Following SCNT, differentiated cells can regain totipotency, thus, cloned animals can be produced. However, this process remains inefficient due to insufficient epigenetic reprogramming, which affects normal embryonic, placental and fetal development [33]. Important events affecting cell reprogramming after SCNT include changes in chromatin accessibility [34], exchange of histone variants [35,36], silencing of genes expressed from the nuclear donor cell [30], DNA demethylation and remethylation [12], as well as changes in histone methylation [37] and histone acetylation patterns [38]. Among the attempts tested to enhance cell reprogramming, increasing histone acetylation has been shown to increase the development of SCNT in several species, including pigs [9,20]. Therefore, treatment with histone deacetylases inhibitors (HDACis), such as Scriptaid and Trichostatin A, has become an integral component of SCNT protocols. While there is consensus that treatment with HDACis promotes chromatin remodeling by facilitating the access of reprogramming factors, it also creates a transcriptional permissive state that may result in the expression of genes that are unnecessary at the early stages of embryo development. Indeed, there is evidence that SCNT embryos retain the transcriptional memory of the nuclear donor cells [27,30,39]. To overcome this undesirable effect of HDACi treatment, this study explored the impact of attenuating both histone acetylation and RNA synthesis on the pre- and post-implantation development of porcine SCNT embryos.

Because previous studies in mice revealed that inhibiting RNA synthesis for a short period following fertilization (from 4 to 20 h), which corresponds to the minor phase of the EGA in the mouse embryo, decreased embryo development [32] our first goal was to test how long RNA synthesis can be inhibited in porcine embryos without affecting their development and quality. We found that RNA synthesis can be inhibited for up to 40 h in both PA and IVF embryos without affecting their development to the blastocyst stage and their total cell number. One interesting observation was that RNA synthesis inhibition for 32 h significantly increased the total cell number in PA-derived but not IVF-derived blastocyst. Recent studies that compared the transcriptional profile of PA and IVF embryos observed more downregulated genes in PA than in IVF embryos (274 vs. 28) having higher developmental capacity (early cleaving embryos) compared to embryos having lower developmental capacity (late cleaving embryos) [40]. Among the downregulated transcripts in the more developmental competent embryos are genes involved in DNA damage response, endoplasmic reticulum stress, apoptosis and autophagy [40]. Therefore, it is worth speculating that DRB treatment may have resulted in PA blastocysts having more cells by attenuating the expression of the genes involved in these pathways.

We then evaluated the impact of preventing RNA synthesis in porcine SCNT embryos treated with the HDACi Scriptaid for 15 h after nuclear transfer. Similarly to PA and IVF embryos, neither development nor blastocyst cell number were affected by inhibiting RNA synthesis for up to 40 h of development in SCNT embryos. Interestingly, the concomitant inhibition of histone deacetylases and transcription for 15 h resulted in SCNT blastocysts having a higher number of cells. The same effect was observed when the transcriptional inhibition was extended to 24 h and 32 h, but not 40 h after nuclear transfer. These findings are in line with our previous observations in bovine SCNT embryos [31] and suggest that normal cell functions are better recapitulated in SCNT embryos during the post-nuclear transfer period when histone deacetylases are inhibited to enhance chromatin reprogramming along with the inhibition of transcription.

To explore the potential consequences of inhibiting RNA synthesis during HDACi treatment on the activation of the transcriptional activity in SCNT embryos, uridine incorporation on D3 of development and qPCR analyses on D2, D3 and D4 of development were performed. These developmental stages encompass the main phase of the embryonic genome activation in the pig embryo, which occurs during the third and fourth cell cycle stages in this species [13,41,42,43]. Moreover, transition from D2 to D4 of development is when pig embryos upregulate the transcription of several genes encoding histone demethylases involved in the regulation of EGA, cell differentiation and genome stability [26,44], some of which were shown to be abnormally expressed in SCNT embryos compared to IVF-derived embryos [13]. By assessing EU incorporation on D3 embryos, we observed an absence of statistical differences in both the proportion of EU positive nuclei and EU fluorescence intensity between embryos treated with Scriptaid alone or both Scriptaid + DRB. This suggests that the onset of the transcriptional activity was not perturbed by DRB treatment. However, quantification of specific transcripts by qPCR revealed that several genes known to be upregulated between D2 and D4 of pig embryo development were more abundantly expressed in embryos treated with Scriptaid + DRB than Scriptaid only. For instance, we observed increased mRNA for *DUXA* on D2, D3 and D4 in DRB-treated embryos. This transcription factor is involved in the regulation of the EGA in mice [45], and a transitory induction of *DUXA* expression in mouse SCNT embryos improved nuclear reprograming, embryo development and cloning efficiency [46]. Similarly, we detected higher mRNA levels for *DPPA2* and *DPPA4* in Scriptaid + DRB-treated embryos than Scriptaid-treated embryos on D3 and D4 of development. *DPPA2* and *DPPA4* are upstream regulators of *DUXA* and are involved in EGA, establishment of cell pluripotency and embryo survival in mice [47,48,49,50]. *DPPA2* and *DPPA4* were shown to be less expressed in mouse SCNT embryos than in fertilized embryos [12], suggesting an improper reprogramming of these genes in SCNT embryos.

Moreover, Scriptaid + DRB-treated embryos expressed more mRNA for *KDM5B* and *KDM7A* on D3 and D4 of development compared to embryos treated with Scriptaid only. These genes were shown to participate in the regulation of EGA, DNA damage response and first cell lineage differentiation in pig embryos [26,44], which suggests these processes are better recapitulated by preventing transcription during deacetylases inhibition early after nuclear transfer in porcine SCNT embryos. In line with this are previous observations that the overexpression of KDM5B reduced the expression of somatic memory genes and improved embryo development and quality in bovine SCNT embryos [39]. Besides regulators of histone modifications, we observed higher mRNA levels for the ubiquitin ligase *RNF114* on D2 and the SUMO-conjugating *UBC9* and SUMO-activating *UBA2* genes on D4 embryos that were treated with Scriptaid + DRB compared with Scriptaid only. These genes were shown to be upregulated during EGA transition and in response to DNA damage induction in pig embryos [51], and supplementation of culture medium with UBA2 improved pig embryo development [52]. In addition, we found that Scriptaid + DRB treatment increased mRNA for *EIF1AX* on D2, D3 and D4 embryos, and *EIF2A* on D4 embryos, which are important markers of EGA in porcine embryos [26,53]. These findings indicate that preventing transcription during deacetylases inhibition improves EGA transition in porcine SCNT embryos by enhancing the expression of important genes involved in the regulation of different cellular pathways.

Finally, to explore if preventing transcription during deacetylases inhibition early after nuclear transfer impacts the post-implantation development of pig SCNT embryos, 1-cell stage embryos derived from three different PFF cultures were transferred to the oviduct of estrus-synchronized gilts. Normal fetal developmental around day 60 of pregnancy and healthy cloned piglets were produced from Scriptaid + DRB-treated embryos, which confirms this treatment can be successfully applied to fully reprogram somatic cells. However, the impact of improving SCNT efficiency need to be further tested, not only by transferring more PPF-derived embryos, but also the effects of using different cell types in pigs and other species.

In conclusion, findings from this study demonstrated that simultaneous exposure of porcine SCNT embryos to Scriptaid, a histone deacetylase inhibitor, and DRB, a reversible transcription inhibitor, for 15 h following nuclear transfer and oocyte activation generated embryos with a higher number of cells, increased transcription of important genes for EGA transition, and enabled full cell reprogramming and creation of healthy cloned piglets. This treatment may facilitate cloning from cells that are more difficult to reprogram, such as more differentiated cells, or cells that are cultured for more passages to enable selection after genetic manipulations; however, this requires further investigation.

## 4. Materials and Methods

### 4.1. Chemicals

Unless otherwise indicated, chemicals and reagents were purchased from Sigma Chemical Company (Sigma-Aldrich; Oakville, ON, Canada).

### 4.2. Collection of Oocytes and In Vitro Maturation

Ovaries from prepubertal gilts were collected from a local abattoir (CBCo Alliance Inc) and transported to the laboratory in saline solution (0.9% NaCl), supplemented with 100 IU/mL penicillin and 10 mg/mL streptomycin, at 30–35 °C. Cumulus-oocyte complex (COCs) were aspirated from follicles with diameters ranging from 3 to 6 mm using a 21 G needle. Only COCs with at least 3 layers of cumulus cells and homogeneous granulated cytoplasm were selected for in vitro maturation (IVM). Groups of 30 COCs were matured in 90 μL drops of maturation medium covered with mineral oil (Fisher Scientific, Ottawa, ON, Canada), in an incubator with an atmosphere of 5% CO_2_ in air, at 38.5 °C. Maturation medium consisted of TCM199 (Life Technologies, Burlington, ON, Canada), supplemented with 5 IU/mL hCG (Chorulon^®^; Intervet Canada Corp, Kirkland, QC, Canada), 10 µg/mL FSH (Folltropin-V^®^; Vetoquinol, Lavaltrie, QC, Canada), 1 mM cyclic adenosine monophosphate (cAMP), 10 ng/mL of epidermal growth factor (EGF; Life Technologies), 100 μg/mL cysteine, 0.91 mM sodium pyruvate, 3.05 mM d-glucose, 20 μg/mL gentamicin (Life Technologies) and 20% porcine follicular fluid. After 22 h, the COCs were washed and transferred to a new drop of IVM media, which was not supplemented with cAMP, hCG and FSH, and cultured for an additional 22–24 h. After approximately 44 h of IVM, cumulus cells were removed by vortexing in TCM199 HEPES-buffered medium supplemented with 0.1% hyaluronidase, and selected oocytes were used for different experiments.

### 4.3. Parthenogenetic Activation

Mature oocytes were activated according to [54]. Briefly, oocytes were exposed to 15 μM Ionomycin for 5 min, then 200 nM TPEN for 15 min, and kept in PZM-3 medium supplemented with 7.5 μg/mL of cytochalasin B for 4 h. After 4 h, oocytes were washed in PZM-3 and placed in culture.

### 4.4. In Vitro Fertilization

IVF was performed in modified Tris-Buffered Medium (mTBM) [55] containing 2 mM caffeine and 0.2% bovine serum albumin (BSA). Groups of 80–100 oocytes were fertilized using 2 × 10^5^ sperm/mL in four-well plates with 500 μL media for 5 h. After 5 h, oocytes were pipetted to remove attached spermatozoa, washed in PZM-3 and placed in culture.

### 4.5. Somatic Cell Nuclear Transfer

For SCNT, mature oocytes were incubated in TCM199 medium supplemented with 0.4 μg/mL demecolcine and 0.05 M sucrose for 1 h. The oocytes were then enucleated in TCM199 HEPES-buffered medium supplemented with 2 mg/mL BSA (fatty acid free), 20 μg/mL gentamicin, and 7.5 μg/mL cytochalasin B (CB) by removing the protruded chromatin and the first polar body using a micropipette attached to a micromanipulator. A nuclear donor cell was transferred into the perivitelline space of each enucleated oocyte. A single DC pulse of 35 V for 50 μs was applied in a 0.28 M mannitol solution, supplemented with 50 μM CaCl_2_, 100 μM MgSO_4_ and 0.1% BSA, to induce oocyte/cell fusion. Oocytes were then transferred to TCM-199 medium supplemented with 2 mg/mL BSA for 1 h to allow cell fusion. After 1 h, oocytes were activated by exposure to 15 μM ionomycin for 5 min followed by 200 nM TPEN for 15 min [54], washed in PZM-3 and placed in culture.

### 4.6. Embryo Culture and Treatments

Embryos were cultured in 60 μL drops of PZM-3 medium supplemented with 3 mg/mL bovine serum albumin (BSA) at 38.5 °C in an atmosphere of 5% CO_2_ in air. Culture medium was supplemented with 10% fetal bovine serum (FBS) on day 5. Embryo cleavage and blastocyst rates were determined at 48 h and 7 days after activation or IVF, respectively. Cleavage rates were calculated based on the number of embryos that initiate cell division (two or more cells) out of the total number of activated or fertilized oocytes. Blastocyst rates were calculated based on the number of embryos that reached the blastocyst stage at D7 of development out of the total number of cleaved embryos. Following PA or IVF, embryos were exposed or not (CT), to 100 µM DRB for different times. Embryos produced by SCNT were all treated with Scriptaid for 15 h (CT) or concomitantly with Scriptaid and DRB for 15 h, and then only DRB for up to 24 h, 32 h, 40 h, 48 h and 72 h following nuclear transfer and oocyte activation. For all three embryo producing methods, the timepoints of DRB treatment were tested in two different experiments due to the large number of oocytes needed for all the groups, with each experiment having its own CT group. The timepoints of exposure to DRB were the following: Experiment 1: 15 h, 24 h, 32 h, and Experiment 2: 40 h, 48 h, 72 h. Depending on the experiment, embryos were used for RNA synthesis assessment, collected at different time points for RNA extraction, or cultured for 7 days, and then those that reached blastocyst stage were fixed and stained for cell counting.

### 4.7. Embryo Transfer

SCNT zygotes were transferred to the oviduct of recipient gilts around 18–20 h following nuclear transfer and activation. In total, 10 gilts were synchronized with oral administration of 20 mg/gilt/day Altrenogest (Regu-mate^®^; Intervet), for 12 days. On the 12th day, gilts received a luteolytic dose of prostaglandin F2 alpha and 750 IU eCG (Novormon; Synthex, Buenos Aires, Argentina) by intramuscular injections. Seventy-two hours later, ovulation was induced with 500 IU hCG (Chorulon^®^) by intramuscular injection. Embryo transfer surgeries were performed around 24 h following hCG injection. Transabdominal ultrasonography was performed between 28–30 days following embryo transfer to determine pregnancy. The transferred SCNT embryos were produced from 3 different porcine fetal fibroblast cultures (PFF A, B and C). Embryos produced with PFF A and B cells were treated with either Scriptaid only or Scriptaid + DRB for 15 h, and two gilts were transferred with embryos from each treatment and cell line. Embryos produced with PFF C cells were all treated with Scriptaid + DRB for 15 h and transferred to two gilts.

### 4.8. Assessment of RNA Synthesis in Porcine Fetal Fibroblasts and Embryos

Detection of RNA synthesis was performed using the Click-iT™ RNA Alexa Fluor™ 594 Imaging Kit (Invitrogen, Life Technologies). SCNT embryos treated or not (CT), with DRB for 15 h following nuclear transfer and oocyte activation, were incubated with 1 mM 5-ethynyl uridine for a total of 8 h, from 68–76 h (end of day 3) or from 92–100 h (end Day 4) after nuclear transfer and oocyte activation. Porcine fetal fibroblasts treated with 100µM DRB for 15 h, 500 nM of Scriptaid for 15 h or 500 nM Scriptaid + 100µM DRB for 15 h, or the absence of any treatment (CT) were incubated with 1 mM 5-ethynyl uridine during the last 2 h of treatment with Scriptaid and/or DRB. After EU exposure, embryos and cells were fixed in 4% paraformaldehyde, stained according to manufacturer’s instructions, mounted on microscope slides, and evaluated to determine RNA synthesis in an epifluorescence microscope (Nikon eclipse 80i—Nikon).

### 4.9. Embryo Staining for Cell Counting

Embryos that reached the blastocyst stage on D7 were fixed in 4% paraformaldehyde for 15 min and then stored in PBS containing 0.3% BSA and 0.1% Triton X-100 at 4 °C. Prior to staining, embryos were incubated at 37 °C for 1 h. Nuclei were stained by exposing the blastocysts to 10 μg/mL 4′,6-diamidino-2-phenylindole (DAPI; Life Technologies) in blocking solution (BSA 3% and Tween 0.2%) for 20 min, mounted on slides using Mowiol, and the number of nuclei was counted in each embryo using an epifluorescence microscope.

### 4.10. RNA Extraction, Reverse Transcription and Quantitative PCR

SCNT embryos were collected on days 2, 3 and 4 of development. Total RNA was extracted from porcine embryos (groups of 20–25) using the PicoPure RNA Isolation Kit (Life Technologies) according to the manufacturer’s instructions. After extraction, RNA was treated with DNase I (Qiagen; Louiville, KY, United States), and then reverse transcribed using the SuperScript VILO cDNA Synthesis Kit (Life Technologies). qRT-PCR reactions were performed in a CFX 384 real-time PCR system (BioRad, Hercules, CA, United States) using the advanced qPCR mastermix (Wisent Bioproducts, St-Bruno, QC, Canada). Primers were designed based on the porcine sequences available in GenBank, and synthesized by IDT (Windsor, ON, Canada) (Table 2). Samples were run in duplicates, and the standard curve method was used to determine the relative abundance of mRNA for each gene. Relative mRNA expression was normalized to the mean abundance of the internal control gene *H2A* [56]. All reactions had efficiency between 90 and 110%, *r*^2^ ≥ 0.98 and slope values from −3.6 to −3.1. Dissociation curve analyses were performed to validate the specificity of the amplified products.

### 4.11. Statistical Analysis

All data were analyzed using the JMP software (SAS Institute Inc., Cary, NC, USA). In each experiment, data were tested for normal distribution with Shapiro–Wilk test. Means were compared using Student’s *t*-test for single comparisons or LSMeans Tukey HSD for multiple comparisons. Results are presented as means ± standard error of the mean (SEM) and *p* < 0.05 was considered statistically different. All experiments were performed at least in three individual replicates.

## Figures and Tables

**Table 1:** Post-implantation development of SCNT embryos produced using three different cell lines and treated with Scriptaid or Scriptaid + DRB for 15 h and transferred to recipient gilts.

Donor Cell	DRB	Gilt (#)	Transferred Embryos (n)	Confirmed Pregnancy (D28-30)	Pregnancy Loss	Fetuses Collected	Piglets Born (n)
PFF A	-	1	174	No	-	-	
	-	2	207	No	-	-	-
	+	3	165	Yes	-	6	-
	+	4	221	Yes	-	4	
PFF B	-	5	89	Yes	Yes	-	-
	-	7	103	Yes	No	-	4
	+	6	90	Yes	No	-	1
	+	8	107	Yes	Yes	-	-
PFF C	+	9	97	Yes	No	-	5
	+	10	102	Yes	No	-	1
Total	-	n = 4	573	2	1	0	4
	+	n = 6	782	6	1	10	7

**Table 2:** Primers used in the quantitative real-time PCR reactions.

Gene	Forward (5′ to 3′)	Reverse (3′ to 5′)	Reference or Accession Number
*DCAF13*	TTCCTTGCTTCCCTGGATGG	CGTACAAAACCTTCATGCGC	[51]
*DNMT1*	ATTCTCTCCTTCGACACGCC	GCCTTTCAGCTCGCCTTTTC	[8]
*DPPA2*	TGAGTACCAGTGGCCAGAAAA	GACTGCAATCTGGTCTCCCA	XM_003358822.4
*DPPA4*	CAGAGACGTTCTTCGGGCTT	TGTGGCAGGGAAGTCTTTTTG	XM_005654065.3
*DUXA*	AGAACACAGACGCAAGCCAA	TAGCTGGTCCGACATCGTCT	XM_021097581.1
*EIF1AX*	ACACCTCCCCGATAGGAGTC	TTGAGCACACTCTTGCCCAT	[26]
*EIF2A*	AGGAGCGTCCTACTATGGGG	TGGCTGGCATGAAGCCATAA	[26]
*H2A*	GGTGCTGGAGTATCTGACCG	GTTGAGCTCTTCGTCGTTGC	[13]
*KDM5B*	GACGTGTGCCAGTTTTGGAC	TCGAGGACACAGCACCTCTA	[13]
*KDM5C*	GGCATGGTCTTCTCAGCCTT	TGAGGGTACCCCATACCAGG	[13]
*KDM6A*	AGCTTTTGTCGAGCCAAGGA	GCATTGGACAAAGTGCAGGG	[13]
*KDM7A*	TCAGGAATAGACGGGCTGGA	TCAGTGCAGTGCAATCAGGT	[13]
*RNF114*	GTCCATAGCACGGACACCAA	CCGACGCTGGATGTGTTCTA	[51]
*UBA2*	GGAGCCGACTTCAAGCAGAT	TGGGGCATCACCAACAACTT	[51]
*UBC9*	CAAGCAGAGGCCTACACGAT	AAGGTCGCTGCTTATGAGGG	[51]

## Supplementary Materials

The supporting information can be downloaded at: https://www.mdpi.com/article/10.3390/ijms232214142/s1.
