# Supplementary material for: Simultaneous Inhibition of Histone Deacetylases and RNA Synthesis Enables Totipotency Reprogramming in Pig SCNT Embryos
Source: Int J Mol Sci. 2022 Nov 16;23(22):14142. doi: 10.3390/ijms232214142 (PMC9697165; doi:10.3390/ijms232214142)
Supplement: Supplementary file 1 [file ijms-23-14142-s001.zip › ijms-1976710-supplementary.pdf]

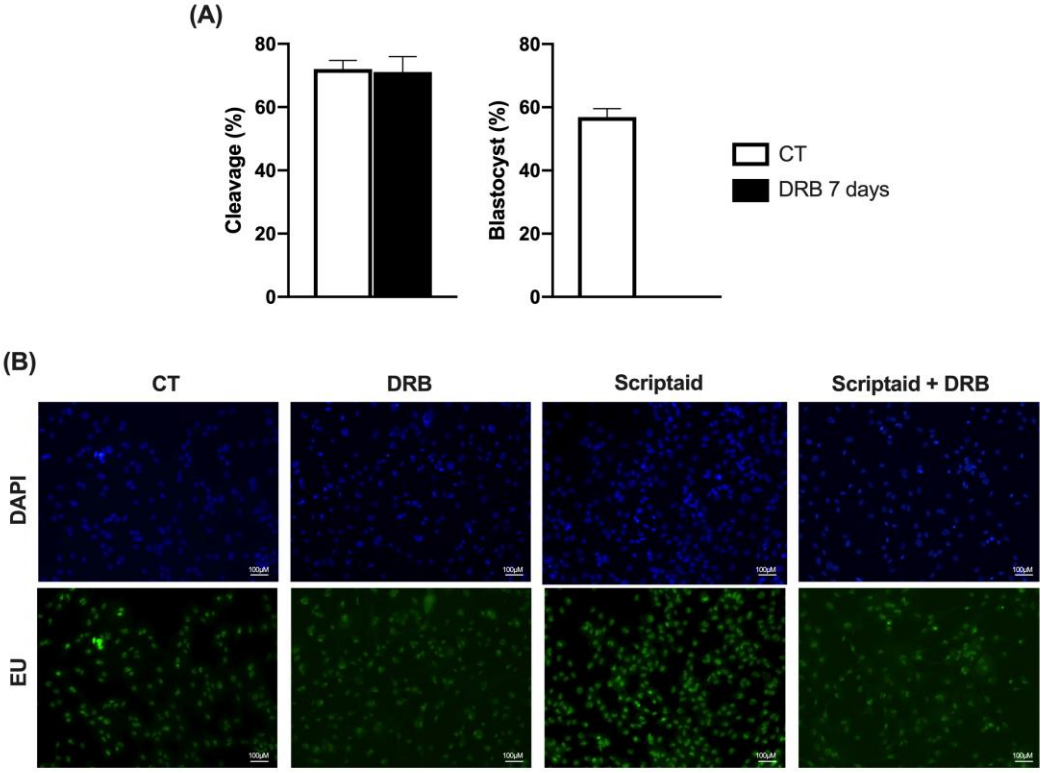

Supplementary Figure S1. Efficacy of transcriptional inhibition by DRB. (A) Cleavage and blastocyst rates of PA embryos that were either not treated (CT, n=90) or treated with DRB (DRB, n=93) for the 7 days of culture. (B) DAPI and EU staining of fibroblast cells that were cultured in control medium only (CT) or in presence of DRB, Scriptaid or Scriptaid + DRB for 15h.
